# Novel Zoonotic Avian Influenza A(H3N8) Virus in Chicken, Hong Kong, China

**DOI:** 10.3201/eid2810.221067

**Published:** 2022-10

**Authors:** Thomas H.C. Sit, Wanying Sun, Anne C.N. Tse, Christopher J. Brackman, Samuel M.S. Cheng, Amy W. Yan Tang, Jonathan T.L Cheung, Malik Peiris, Leo L.M. Poon

**Affiliations:** Government of the Hong Kong Special Administrative Region, Hong Kong, China (T.H.C. Sit, A.C.N. Tse, C.J. Brackman);; The University of Hong Kong, Hong Kong (W. Sun, S.M.S. Cheng, A.W. Yan Tang, J.T.L. Cheung, M. Peiris, L.L.M. Poon)

**Keywords:** Avian influenza virus, influenza virus, viruses, H3N8, influenza, emergence, human, serology, immunity, population, novel, respiratory infections, chicken, zoonoses, Hong Kong, China

## Abstract

Zoonotic and pandemic influenza continue to pose threats to global public health. Pandemics arise when novel influenza A viruses, derived in whole or in part from animal or avian influenza viruses, adapt to transmit efficiently in a human population that has little population immunity to contain its onward transmission. Viruses of previous pandemic concern, such as influenza A(H7N9), arose from influenza A(H9N2) viruses established in domestic poultry acquiring a hemagglutinin and neuraminidase from influenza A viruses of aquatic waterfowl. We report a novel influenza A(H3N8) virus in chicken that has emerged in a similar manner and that has been recently reported to cause zoonotic disease. Although they are H3 subtype, these avian viruses are antigenically distant from contemporary human influenza A(H3N2) viruses, and there is little cross-reactive immunity in the human population. It is essential to heighten surveillance for these avian A(H3N8) viruses in poultry and in humans.

Diverse influenza A viruses are found in aquatic waterfowl, poultry, swine, horses, aquatic mammals, bats, and domestic pets such as cats and dogs. Although there is a diversity of virus hemagglutinin (H1–H16) and neuraminidase (N1–N9) subtypes in aquatic birds, more restricted numbers of virus subtypes are established in other species, including chicken (*1*). The high mutation rates associated with an error-prone virus replication complex and the presence of a segmented genome enables genetic reassortment of gene segments of viruses of different species and interspecies transmission and adaptation to new hosts.

Influenza A virus subtypes H9 and H6 have formed established lineages in domestic chicken and game birds (quail, pheasant) farmed for consumption in Asia (*2*). The internal gene constellation of H9N2 viruses contains hemagglutinin (HA) and neuraminidase (NA) genes acquired from aquatic waterfowl to generate H5N1, H5N6, H7N9, and H10N8 viruses through genetic reassortment, and many of these viruses also became established in poultry, subsequently posing zoonotic and pandemic threats (*3*–*5*). A novel influenza A(H3N8) virus has been recently reported to cause zoonotic infection in Henan Province, China (*6*).

In this context, we report detection of novel H3N8 viruses recently identified in chicken in live poultry markets and chicken farms in Hong Kong, China, that are genetically similar to the zoonotic H3N8 viruses reported in mainland China (*6*). We also report that these recent H3N8 viruses have arisen in a manner akin to zoonotic H5N1, H7N9, and H10N8 viruses and that there is little cross-reactive immunity in the human population to these chicken H3N8 viruses.

## Methods

### Influenza A Virus Surveillance and Virologic Testing of Poultry Farms

The Department of Agriculture, Fisheries and Conservation in Hong Kong routinely conducts virologic surveillance on each batch of chickens from local farms before release for sale. The surveillance is conducted on 30 unvaccinated sentinel chickens cohoused with each chicken flock. During December 14, 2021‒January 21, 2022, we obtained oropharyngeal and cloacal swab samples from 30 chickens on each of 28 poultry farms. We combined samples into pools of 6 and placed each pool into a vial of virus transport medium (medium 199 plus antimicrobial drugs).

In a follow-up investigation of 4 farms found positive for H3N8 virus, we conducted more intensive surveillance during May 2022 to check for any continuing evidence of on-farm viral circulation. We sampled a total of 50 chickens by using oropharyngeal and cloacal swabbing, again in pools containing 6 specimens.

### Influenza A Surveillance in Live Poultry Markets

The School of Public Health of The University of Hong Kong routinely conducts surveillance in live poultry market stalls in Hong Kong (n = 116) by sampling from each stall fecal droppings (n = 10), drinking water in poultry cages (n = 2), and chopping boards and the inner wall and outer surface of defeathering machines used (n = 3) in preparation of slaughtered poultry for sale (*7*). All 116 poultry stalls were sampled every 3 months. Samples were individually collected and placed into vials of virus transport medium (medium 199 plus antimicrobial drugs), and samples were kept in cool packs for transport to the laboratory.

### Real-Time Reverse Transcription PCR for Detection of Influenza A Viruses

We extracted viral RNA from chicken oropharyngeal and cloacal swab specimens by using the MagNA Pure 96 DNA and Viral NA Small Volume Kit (Roche, https://lifescience.roche.com) according to the manufacturer’s instructions. We tested eluted specimen RNA by using real-time reverse transcription PCR (RT-PCR) for the influenza A virus matrix (M) gene as described (*8*).

We tested swab specimen supernatants of all influenza A virus M gene‒positive swab specimens by RT-PCR for H5, H7, and H9 (*9*) and for virus isolation. We identified virus subtype of M gene‒positive swab specimens negative for H5, H7, and H9 by using genetic sequencing of the virus isolate or directly from the swab specimen.

### Virus Isolation

We inoculated 0.2 mL of swab specimen supernatant of all influenza A virus M gene‒positive swab specimens into the allantoic cavity of three 9–11-day-old specific pathogen–free embryonated eggs and incubated at them at 36°C (± 2°C) for 4 days. We candled the eggs daily, and harvested allantoic fluid. We subtyped virus isolates by using hemagglutination inhibition (HI) tests and reference panels of antiserum to a range of influenza virus A subtypes (*7*).

### Genetic Sequencing of Virus Isolates and Phylogenetic Analysis

We deduced near full-length genomes from virus grown in allantoic fluid samples by using an Illumina Sequencing Protocol (https://www.illumina.com) as described (*5**,**10**–**12*). We removed low-quality base pairs in the raw data by using Fastp (*13*) and selected reference sequence by using SPAdes (*11*) and BLAST (*14*). We generated consensus sequences by using BWA (https://arxiv.org/abs/1303.3997) and Pilon (*15*) and aligned sequences by using MUSCLE (*16*) and public sequences from GenBank and GISAID (https://www.gisaid.org) (Appendix Table). We constructed phylogenic trees by using IQtree (*17*) with the general time-reversible plus gamma model and 1,000 bootstrap replicates.

### DNA Bar Coding

We conducted PCR amplification of the mitochondrial cytochrome oxidase I gene for host-species-identification as described (*18*). We sequenced the amplified ≈700-bp PCR fragment of the cytochrome oxidase I gene by using the 3730xl DNA Analyzer (Applied Biosystems, https://www.thermofisher.com) and analyzed by using the barcoding software bold, which provides a taxonomic assignment to the query sequence by using a linear search to collect nearest neighbors (lowest percentage divergence) from a global alignment of all reference sequences (*19*).

### Serologic Analysis

We used the HI test to detect the seroprevalence to 1 of the novel H3N8 viruses, A/chicken/Hong Kong/MKT-AB13cp/2022, and human seasonal virus A/Switzerland/8060/2017 (H3N2) in a panel of age-stratified blood donor serum samples collected during 2019–2020. The study protocol was approved by the University of Hong Kong. We also tested HI titers of a World Health Organization reference antiserum to A/Switzerland/8060/2017 against A/chicken/MKT-AB13cp/2020 H3N8 virus (original serum dilution provided was 1:128) in comparison with the homologous virus A/Switzerland/8060/2017. The HI tests were conducted as described (*20**,**21*). We analyzed the effect of age-stratified seroprevalence on the reproduction number (R_0_) and population immunity as described (*22*).

## Results

During routine virologic surveillance on chicken farms, H3N8 viruses were first identified on samples collected from 2 broiler farms (farms A and B) in December 2021 and subsequently detected on 2 other broiler farms in January 2022 (farms C and D) (Table 1). On 1 of the farms (B), H3N8 virus was detected on 3 other occasions during February and March 2022.

**Table 1:** Virologic results for local farm chickens positive for avian influenza A(H3N8) virus under active surveillance, Hong Kong, China*

Farm	Date sample collected	No. vials	No. (%) positive by RT-PCR			No. (%) positive by virus isolation	
			H9	H3		H9	H3
A	2021 Dec 14	10	0	6 (60)		0	6 (60)
B	2022 Dec 28	10	0	6 (60)		0	3 (30)
	2022 Feb 21	10	0	0		0	1 (10)
	2022 Mar 7	10	0	6 (60)		0	5 (50)
	2022 Mar 21	10	0	1 (10)		0	1 (10)
C	2022 Jan 12	10	0	6 (60)		0	6 (60)
D	2022 Jan 21	10	0	6 (60)		0	6 (60)

*No other influenza virus subtypes were detected. RT-PCR, reverse transcription PCR.

Of the 4 chicken farms that had positive virologic results, all had serologic evidence (HI titers >16) of past influenza A(H3N8) virus infection; 3 of 4 farms had >26 of 30 birds sampled on each farm in February 2022 test serologically positive (Table 2). Chicken producers were subsequently advised to conduct thorough disinfection and strengthen farm biosecurity to prevent further spread and eliminate the virus.

**Table 2:** Retrospective seroprevalence of antibodies to A/chicken/Hong Kong/22-10782/2022 influenza A(H3N8) virus in chicken serum samples collected from affected farms, Hong Kong, China*

Farm	Date samples collected	No.	H3N8 HI titer >1:16, no. (%)	H3N8 GMT (95% CI)
A	2022 Feb 16	30	26 (86.7)	28.51 (20.01–40.61)
B	2022 Feb 9	30	2 (6.7)	1.35 (0.96–1.91)
C	2022 Feb 16	30	29 (96.7)	46.31 (31.97–67.09)
D	2022 Feb 24	30	26 (86.7)	16 (10.25–24.99)

*Serologic study was conducted on 28 farms in January‒February 2022. Only data for 4 farms positive for H3N8 virus are shown. GMT, geometric mean titer; HI, hemagglutination inhibition.

Follow-up virologic testing in May 2022 of 150 chickens from each of the 4 positive farms yielded negative results (Table 3). As of the end of June 2022, there has been no additional detection of H3N8 on any farms.

**Table 3:** Follow-up virologic results for local chicken farms previously positive for avian influenza A(H3N8) virus, Hong Kong, China*

Farm	Date samples collected	No. samples†	No. (%) positive by RT-PCR			No. (%) positive by virus isolation	
			H9	H3		H9	H3
A	2022 May 10	50	0	0		0	0
B	2022 May 11	50	0	0		0	0
C	2022 May 10	50	2 (4)	0		0	0
D	2022 May 11	50	0	0		0	0

*RT-PCR, reverse transcription PCR. †Includes oropharyngeal and cloacal samples.

During January 2022–June 2022, we collected and tested 3,525 environmental swab samples of fecal droppings, drinking water in poultry cages, and chopping boards and defeathering machines in live poultry markets and stalls sampled (Table 4). An environmental swab specimen collected from a chicken defeathering machine on January 12 and a swab specimen collected from a poultry chopping board on January 20 from 2 different live poultry markets were positive for influenza A(H3N8) viruses. The second market only sells chicken, and the first market additionally sells chilled dressed duck slaughtered elsewhere. The species of origin from both swab specimens was determined by DNA bar coding to be domestic chicken (*Gallus domesticus*).

**Table 4:** Samples tested in live poultry markets and those positive for influenza A virus, Hong Kong, China, 2022*

Month	No. swabs tested	No. markets sampled	No. stalls sampled	No. (%) positive by RT-PCR				No. (%) positive by virus isolation		
				H9	H6	H3		H9	H6	H3
Jan	555	25	37		0	2 (0.36)		0	0	2 (0.36)
Feb	435	16	29	6 (1.38)	2 (0.46)	0		4 (0.92)	1 (0.23)	0
Mar	705	34	47	4 (0.57)	0	0		1 (0.14)	0	0
Apr	585	27	39	2 (0.34)	0	0		0	0	0
May	630	25	42	0	0	0		0	0	0
Jun	615	32	41	2 (0.33)	0	0		2 (0.33)	0	0
Total	3,525	159	235	14 (0.40)	2 (0.06)	2 (0.06)		7 (0.20)	1 (0.03)	2 (0.06)

*No other influenza A virus subtypes were detected. RT-PCR, reverse transcription PCR

We sequenced 7 H3N8 viruses in this study and submitted them to GISAID (Table 5). Phylogenetic analysis of the full-genome sequence of poultry H3N8 viruses showed that the chicken H3N8 viruses from farms and poultry markets are closely related to each other and to an H3N8 virus associated with zoonotic disease in mainland China (Figure; Appendix Figure). The polymerase basic 1, polymerase basic 2, polymerase acidic, NA, nonstructural protein, and M gene segments were derived from the G57 sublineage of influenza A(H9N2) viruses commonly found in mainland China (*23*), whereas the HA gene sequences belong to the Eurasian avian H3 lineage, which has been detected in ducks and other wild birds (*24*).

**Table 5:** Influenza A (H3N8) viruses genetically sequenced in from chicken farms, live poultry markets, and the Mai Po Wetlands, Hong Kong, China

Virus name	Date of collection	Place and site of collection	DNA barcoding	GISAID accession no.*
A/chicken/Hong Kong/21-17040/2021 (H3N8)	2021 Dec 13	Farm A	Not relevant	ON909094-ON909101
A/chicken/Hong Kong/21-17632/2021 (H3N8)	2021 Dec 28	Farm B	Not relevant	ON909102-ON909109
A/Env/Hong_Kong/MKT_TYEB_13d2/2022 (H3N8)	2022 Jan 12	Live poultry market, defeathering machine	*Gallus gallus*	EPI_ISL_13566013
A/Env/Hong_Kong/MKT_AB_13cp/2022 (H3N8)	2022 Jan 20	Live poultry market, chopping board	*Gallus gallus*	EPI_ISL_13566014
A/Env/HongKong/MP16_1834/2016 (H3N8)	2016 Dec 21	Mai Po wetlands	*Anas acuta*	EPI_ISL_13566015
A/Env/HongKong/MP18_0131/2018 (H3N8)	2018 Nov 14	Mai Po Wetlands	*Anas clypeata*	EPI_ISL_13566016
A/Env/HongKong/MP18_0135/2018 (H3N8)	2018 Nov 14	Mai Po Wetlands	*Anas clypeata*	EPI_ISL_13566017

*GISAID, https://www.gisaid.org.

**Figure:**
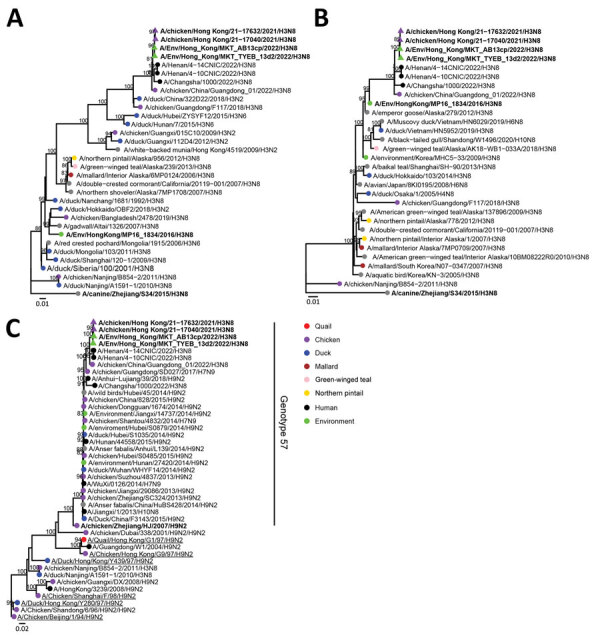
Phylogenetic analysis of influenza A(H3N8) viruses isolated from chicken farms, live poultry markets, and the Mai Po Wetlands, Hong Kong, China (bold). A) Hemagglutinin gene segment; B) neuraminidase gene segment; C) polymerase basic 2 gene segments. Strains listed in Table 5 were analyzed with other relevant virus sequence data available in public databases (accession numbers in Appendix Table). Trees were generated by using IQ-tree (https://www.iqtree.org) with the general time reversible plus gamma model. Bootstrap values >80% are shown. Scale bars indicate estimated genetic distances.

The NA gene sequences of the poultry A(H3N8) viruses belonged to the North American lineage, but a closely related N8 NA sequence had previously been detected in A/Env/Hong_Kong/MP16_1834/2016 (H3N8), a virus isolated on December 21, 2018, from the Mai Po Wetlands, Hong Kong, in 2018, obtained from a fecal specimen identified by DNA bar coding to be derived from a Northern pintail duck (*Anas acuta*) (Table 5). Two other H3N8 viruses isolated from fecal droppings collected from the Mai Po Wetlands on November 14, 2018, identified to be from a Northern shoveler duck (*Anus clypeata*) were genetically unrelated in all gene segments to the chicken H3N8 viruses. The N8 gene segment sequence also is closely related to other aquatic wild bird H3 viruses from mainland China. Other than for the N8 NA gene segment, none of the other gene segments of the poultry H3N8 viruses were derived from the wild bird H3N8 viruses detected in the Mai Po Wetlands of Hong Kong. These viruses were distinct from chicken H3N8 viruses previously reported in mainland China (*25*). However, 1 sequence of a virus from chicken similar in all 8 gene segments to our Hong Kong H3N8 viruses is available in virus genetic sequence databases (Figure).

The HI titer of the World Health Organization reference antiserum to human seasonal H3N2 virus A/Switzerland/8060/2017 against the homologous virus antigen was 1:128, and the titer against A/chicken/Hong Kong/MKT-AB13cp/2022 was <1:10, suggesting limited antigenic cross-reactivity of current human seasonal H3N2 viruses with these novel avian H3N8 viruses. The overall seroprevalence (HI titer >1:40) to A/chicken/Hong Kong/MKT-AB13cp/2022 (H3N8) in age-stratified human serum samples was 3.2% (Table 6). In contrast, as expected, we found high (58.7%) seroprevalence to a recent human seasonal A/Switzerland/8060/2017 (H3N2) virus in this same panel of serum samples.

**Table 6:** Seroprevalence of antibodies to human seasonal influenza virus A/Switzerland/8060/2017 (H3N2) and A/chicken/Hong Kong/MKT-AB13cp/2022 (H3N8) virus in age-stratified human serum samples from blood donors, Hong Kong, China, 2020*

Age group, y	No.	H3N2 HI titers, no. (%)			H3N8 HI titer, no. (%)		H3N2 GMT (95% CI)	H3N8 GMT (95% CI)
		>1:10	>1:40		>1:10	>1:40		
10–19	10	10 (100)	9 (90)		0	0	183.8 (74.8–451.7)	5 (5–5)
20–29	10	7 (70)	7 (70)		0	0	37.32 (11.8–118.6)	5 (5–5)
30–39	10	5 (50)	5 (50)		0	0	20 (6.7–59.9)	5 (5–5)
40–49	10	8 (80)	6 (60)		3 (30)	1 (10)	30.31 (12.6–73.1)	7.071 (4.4–11.5)
50–59	10	3 (30)	0		2 (20)	0	7.1 (4.6–10.8)	5.743 (4.7–7.1)
60–69	10	9 (90)	9 (90)		1 (10)	1 (10)	56.6 (25.8–123.9)	6.156 (3.9–9.9)
70–79	3	1 (33)	1 (3)		0	0	12.6 (0.2–671.9)	5 (5–5)
Total	63	43 (68.3)	37 (58.7)		6 (9.5)	2 (3.2)	32.8 (22.1–48.8)	5.6 (5.1–6.2)

*GMT, geometric mean titer; HI, hemagglutination inhibition.

Human population immunity to a potentially zoonotic virus is a major parameter that is included in the risk assessment of animal viruses for a pandemic threat. We have described an approach to assess that risk by estimating the effect of age-stratified immunity in the human population by using HI tests on R_0_ of such a virus if it were to become transmissible in humans (*22*). We found that the observed seroprevalence in humans would provide little or no resistance to such a virus, if it were to acquire other factors required for transmission between humans (Table 7).

**Table 7:** Estimates of effect of observed seroprevalence on human population immunity and reproductive numbers needed to cause a pandemic for novel zoonotic avian influenza virus A(H3N8) virus in chicken, Hong Kong, China*

Virus used	Estimate (95% CI)		
	Proportion of population immune	Relative reduction in reproduction number	Smallest reproductive number needed to cause a pandemic
A/Switzerland/8060/2017(H3N2)	0.393 (0.337–0.446)	0.375 (0.317–0.43)	1.601 (1.464–1.755)
A/chicken/Hong Kong/MKT0AB13cp.2022 (H3N8)	0.029 (0.012–0.058)	0.032 (0.013–0.061)	1.033 (1.013–1.066)

*See Nguyen et al. (*17*) for the methods used.

## Discussion

We report detection of chicken influenza A(H3N8) viruses from live poultry markets and farms in Hong Kong. These viruses were genetically similar to each other and to a recently reported zoonotic H3N8 virus in mainland China (*6*). The viruses were novel reassortants that have virus internal gene segments derived from H9N2 lineage genotype 57 viruses (A/chicken/Zhejiang/HJ/2007-like) established in poultry in mainland China, but the H3 and N8 gene segments were derived from wild aquatic bird influenza A viruses. The H9N2 virus internal gene cassette was previously reported to facilitate the emergence of reassortant influenza A viruses of zoonotic potential (*26*). These chicken H3N8 viruses in Hong Kong were distinct from H3N8 viruses reported from poultry in mainland China (*25*), but a A/chicken/China/Guangdong_01/2022 (H3N8) virus genetically similar to these viruses in all 8 gene segments is reported in public databases (Appendix Table). These H3N8 viruses were also distinct from H3N8 viruses reported in horses, dogs and cats (*27**–**29*).

These novel H3N8 viruses appear to have arisen in a manner analogous to the emergence of previous zoonotic H7N9 and H10N8 viruses, in which the H9N2 viruses enzootic in chicken and other game birds in China acquired HA and NA gene segments from wild, aquatic bird viruses. Wild aquatic birds share ecosystems with domestic ducks, and it is inevitable that influenza viruses will also be shared in such ecosystems. Subsequent trade systems in which domestic ducks and chickens (and other game birds) are mixed in close proximity within wholesale and retail poultry markets provide the opportunity for H9N2 viruses in chicken to acquire HA and NA gene segments from domestic ducks, as has been postulated in the emergence of H7N9 and H10N8 viruses (*4*).

Pandemics emerge when influenza viruses of birds, swine, or other mammals adapt to transmission between humans and when the human population lacks immunity to the hemagglutinin of the newly emerged virus. Cross-reactive immunity in humans is 1 parameter that is considered when risk assessing the pandemic threat from a newly emerged animal influenza virus (*30*). Our data suggest that there is little antigenic cross-reactivity between contemporary seasonal H3N2 viruses and the H3N8 virus. The overall HI test seroprevalence at a titer >1:40 to H3N8 in age-stratified serum samples collected from blood donors in Hong Kong was 3.2%, and the estimated proportion of the population immune (weighted for age structure) was 2.9% (95% CI 1.2%–5.8%). We estimated that if this H3N8 virus acquired transmissibility between humans and acquired an R_0_ >1.033, cross-reactive population immunity would fail to impede its onward transmission in the human population. For comparison, similar estimation of the minimal R_0_ required for the 2009 pandemic H1N1 virus to spread in face of population immunity before its emergence and spread in 2009 was 1.231 (95% CI 1.185–1.292), a markedly higher threshold to cross (*22*).

In conclusion, we report the emergence of a novel influenza A(H3N8) virus in chickens in Hong Kong. This virus might have major zoonotic and pandemic potential. Our results indicate the need to enhance surveillance for this virus in poultry, carry out comprehensive risk assessment of such a virus, and prepare pandemic seed vaccine strains if justified by such risk assessment.

Appendix Additional information on novel zoonotic avian influenza A(H3N8) virus in chicken, Hong Kong, China.
