# Supplementary material for: Novel Zoonotic Avian Influenza A(H3N8) Virus in Chicken, Hong Kong, China
Source: Emerg Infect Dis. 2022 Oct;28(10):2009–15. doi: 10.3201/eid2810.221067 (PMC9514342; doi:10.3201/eid2810.221067)
Supplement: Appendix — Additional information on novel zoonotic avian influenza A(H3N8) virus in chicken, Hong Kong, China. [file 22-1067-Techapp-s1.pdf]

Article DOI: https://doi.org/10.3201/eid2810.221067

Novel Zoonotic Avian Influenza A(H3N8) Virus in Chicken, Hong Kong, China

Appendix

Appendix Table. Virus gene accession numbers of sequences used in phylogenetic trees, Hong Kong, China*

Gene	Identification	GISAID accession no.
HA	A/canine/Zhejiang/S34/2015	EPI1226271
HA	A/Changsha/1000/2022	EPI2035832
HA	A/chicken/Bangladesh/2478/2019	EPI1888010
HA	A/chicken/China/Guangdong_01/2022	ON626399.1
HA	A/chicken/Guangdong/F117/2018	EPI1489636
HA	A/chicken/Guangxi/015C10/2009	KT022237.1
HA	A/chicken/Nanjing/B854-2/2011	KU158890
HA	A/double-crested cormorant/California/20119-001/2007	CY075949.1
HA	A/duck/China/322D22/2018	MN443576.1
HA	A/duck/Guangxi/112D4/2012	KT022269.1
HA	A/duck/Hokkaido/OBF2/2018	LC496328.1
HA	A/duck/Hubei/ZYSYF12/2015	KY415604.1
HA	A/duck/Hunan/7/2015	KX121270.1
HA	A/duck/Mongolia/103/2011	LC339755.1
HA	A/duck/Nanchang/1681/1992	CY006016.1
HA	A/duck/Nanjing/A1591-1/2010	KU158889
HA	A/duck/Shanghai/120-1/2009	EPI774951
HA	A/duck/Siberia/100/2001	AB450457.1
HA	A/gadwall/Altai/1326/2007	CY049804.1
HA	A/green-winged teal/Alaska/239/2013	KY130977.1
HA	A/Henan/4-10CNIC/2022	EPI2026165
HA	A/Henan/4-14CNIC/2022	EPI2026173
HA	A/mallard/Interior Alaska/6MP0124/2006	CY078875.1
HA	A/northern pintail/Alaska/956/2012	KY130961.1
HA	A/northern shoveler/Alaska/7MP1708/2007	CY045439.1
HA	A/red crested pochard/Mongolia/1915/2006	GQ907326.1
HA	A/white-backed munia/Hong Kong/4519/2009	AB557631.1
M	A/chicken/Anhui/AH196/2015	MN135864.1
M	A/chicken/China/E743/2014	MN100789.1
M	A/chicken/China/Guangdong_01/2022	ON626400.1
M	A/chicken/Fujian/3.15_FZHX0029-O/2018	MW101045.1
M	A/chicken/Guangdong/12.29_SZBJ007-O/2016	MW103691.1
M	A/chicken/Guangxi/C227/2015	KX130848.1
M	A/chicken/Hainan/1.14_HKPL001-O/2018	MW101314.1
M	A/chicken/Hebei/BD2/2016	OM018678.1
M	A/Chicken/Hong Kong/G9/97	AF156416
M	A/Chicken/Hong Kong/G9/97	AF156472
M	A/chicken/Hubei/2014	KT164854.1
M	A/chicken/Nanjing/B854-2/2011	KU158897
M	A/chicken/Shaanxi/xa0414/2013	KM609625.1
M	A/Chicken/Shandong/6/96	EPI81796
M	A/Chicken/Shanghai/F/98	AY253751
M	A/chicken/Tianjin/614/2012	KF059287.1
M	A/chicken/Wuhan/JXQL01/2015	KU143327.1
M	A/Duck/China/F1053/2015	MN100837.1
M	A/Duck/Hong Kong/Y280/97	AF156475
M	A/duck/Nanjing/A1591-1/2010	KU158896
M	A/goose/China/146G30/2013	MN865901.1
M	A/Hong_Kong/3239/2008	CY055159.1
M	A/Jiangxi/1/2013	KM392414.1
M	A/pigeon/Fujian/3.15_FZHX0014-C/2018	MW101306.1
M	A/pigeon/Guizhou/11.30_ZYLJJ021-C/2016	MW110331.1

Gene	Identification	GISAID accession no.
M	A/Quail/Hong Kong/G1/97	AF156477
M	A/Anhui-Lujiang/39/2018	EPI1315829
M	A/Changsha/1000/2022	EPI2035839
M	A/chicken/Dubai/338/2001	EPI110286
M	A/chicken/Guangxi/DX/2008	EPI610276
M	A/chicken/Zhejiang/HJ/2007	EPI221856
M	A/Duck/Hong_Kong/Y439/97	EPI6061
M	A/Environment/Jiangxi/14737/2014	EPI858161
M	A/Guangdong/W1/2004	EPI846050
M	A/Henan/4-10CNIC/2022	EPI2026162
M	A/Henan/4-14CNIC/2022	EPI2026170
M	A/Hunan/44558/2015	EPI680521
NA	A/American green-winged teal/Alaska/137896/2009	KX714444.1
NA	A/American green-winged teal/Interior Alaska/10BM08222R0/2010	CY143558.1
NA	A/aquatic bird/Korea/KN-3/2005	EU301277.1
NA	A/avian/Japan/8KI0195/2008	CY079213.1
NA	A/baikal teal/Shanghai/SH-90/2013	KJ907545.1
NA	A/black-tailed gull/Shandong/W1496/2020	OM373216.1
NA	A/canine/Zhejiang/S34/2015	EPI1226273
NA	A/Changsha/1000/2022	EPI2035833
NA	A/chicken/China/Guangdong_01/2022	ON626401.1
NA	A/chicken/Guangdong/F117/2018	EPI1489637
NA	A/chicken/Nanjing/B854-2/2011	KU158904
NA	A/double-crested cormorant/California/20119-001/2007	CY075951.1
NA	A/duck/Hokkaido/103/2014	LC339749.1
NA	A/duck/Nanjing/A1591-1/2010	KU158903
NA	A/duck/Osaka/1/2005	AB472032.1
NA	A/duck/Vietnam/HN5952/2019	MW935355.1
NA	A/emperor goose/Alaska/279/2012	KY130789.1
NA	A/environment/Korea/MHC5-33/2009	JN087354.1
NA	A/green-winged teal/Alaska/AK18-WB1-033A/2018	MN988080.1
NA	A/Henan/4-10CNIC/2022	EPI2026163
NA	A/Henan/4-14CNIC/2022	EPI2026171
NA	A/mallard/Interior Alaska/7MP0709/2007	CY045433.1
NA	A/mallard/South Korea/N07-0347/2007	MN530633.1
NA	A/Muscovy duck/Vietnam/HN6029/2019	MW873237.1
NA	A/northern pintail/Alaska/778/2012	KY130901.1
NA	A/northern pintail/Interior Alaska/1/2007	CY039780.1
NP	A/Anhui-Lujiang/39/2018	EPI1315827
NP	A/Changsha/1000/2022	EPI2035837
NP	A/Chicken/Beijing/1/94	AF156423
NP	A/chicken/China/F998/2015	MN100542.1
NP	A/chicken/China/Guangdong_01/2022	ON626402.1
NP	A/chicken/Dubai/338/2001	EPI110332
NP	A/chicken/Fujian/9.24_FZHX0073-C/2018	MW103321.1
NP	A/chicken/Ganzhou/GZ50/2015	KY415778.1
NP	A/chicken/Guangxi/C227/2015	KX130843.1
NP	A/chicken/Guangxi/DX/2008	EPI610279
NP	A/Chicken/Hong Kong/G9/97	AF156444
NP	A/chicken/Jiangxi/5.26_NCNP5Q2-OC/2017	MW106449.1
NP	A/chicken/Nanjing/B854-2/2011	KU158911
NP	A/chicken/Shaanxi/xa0414/2013	KM609705.1
NP	A/Chicken/Shandong/6/96	EPI81909
NP	A/chicken/Vietnam/H7F-14-BN4-437/2014	MH560138.1
NP	A/chicken/Zhejiang/HJ/2007	EPI221852
NP	A/Duck/China/F3143/2015	MN100556.1
NP	A/duck/Fujian/1.25_FZHX0022-C/2018	MW099128.1
NP	A/Duck/Hong Kong/Y280/97	AF156419
NP	A/Duck/Hong_Kong/Y439/97	EPI5945
NP	A/duck/Japan/AQ-HE5/2015	LC208505.1
NP	A/duck/Jiangxi/09.22_NCDZT43C2-O/2016	MW098624.1
NP	A/duck/Jiangxi/4.30_NCNP96N2-OC/2017	MW108841.1
NP	A/duck/Nanjing/A1591-1/2010	KU158910
NP	A/Environment/Jiangxi/14737/2014	EPI858159
NP	A/goose/China/146G30/2013	MN865899.1
NP	A/Guangdong/MZ058/2016	KX808589.1
NP	A/Guangdong/W1/2004	EPI846048

Gene	Identification	GISAID accession no.
NP	A/Henan/4-10CNIC/2022	EPI2026164
NP	A/Henan/4-14CNIC/2022	EPI2026172
NP	A/Hong_Kong/3239/2008	CY055157.1
NP	A/Hunan/44558/2015	EPI680519
NP	A/Jiangxi/1/2013	KM392412.1
NP	A/pigeon/Fujian/3.15_FZHX0008-C/2018	MW099166.1
NP	A/Quail/Hong Kong/G1/97	AF156407
NP	A/Quail/Hong Kong/G1/97	AF156421
NS	A/Anhui-Lujiang/39/2018	EPI1315826
NS	A/Changsha/1000/2022	EPI2035838
NS	A/Chicken/Beijing/1/94	AF156409
NS	A/chicken/China/63/2019	MN263214.1
NS	A/chicken/China/Guangdong_01/2022	ON626403.1
NS	A/chicken/Daye/DY0602/2017	MF795003.1
NS	A/chicken/Dubai/338/2001	EPI110370
NS	A/chicken/Fujian/6.22_FZHX0627-O/2017	MW103180.1
NS	A/chicken/Ganzhou/GZ86/2016	KY415947.1
NS	A/chicken/Guangxi/DX/2008	EPI610275
NS	A/chicken/Hebei/BD2/2016	OM019055.1
NS	A/chicken/Hebei/HS8/2019	OM019059.1
NS	A/chicken/Hunan/1.17_YYGKK200-OC/2017	MW104916.1
NS	A/chicken/Jiangxi/10.31_NCDZT019-O/2018	MW105828.1
NS	A/chicken/Nanjing/B854-2/2011	KU158918
NS	A/chicken/Ningxia/12.01_XXYN012-O/2016	MW106636.1
NS	A/chicken/Shandong/3424/2016	MH667576.1
NS	A/Chicken/Shandong/6/96	EPI81985
NS	A/Chicken/Shanghai/F/98	AY253752
NS	A/Chicken/Shanghai/F/98	AY253753
NS	A/Chicken/Shanghai/F/98	AY253755
NS	A/Chicken/Shanghai/F/98	AY253756
NS	A/chicken/Taishun/TS2/2016	KY415941.1
NS	A/chicken/Zhejiang/HJ/2007	EPI221857
NS	A/Duck/Hong_Kong/Y439/97	EPI6103
NS	A/duck/Hunan/5.24_YYGK93P2-OC/2017	MW108428.1
NS	A/duck/Nanjing/A1591-1/2010	KU158917
NS	A/Environment/Jiangxi/14737/2014	EPI858160
NS	A/Guangdong/W1/2004	EPI846051
NS	A/Henan/4-10CNIC/2022	EPI2026161
NS	A/Henan/4-14CNIC/2022	EPI2026169
NS	A/Hong_Kong/3239/2008	CY055160.1
NS	A/Hunan/44558/2015	EPI680520
NS	A/Jiangxi/1/2013	KM392415.1
PA	A/Anhui-Lujiang/39/2018	EPI1315825
PA	A/Athene noctua/Tianjin/Y14/2017	MH114057.1
PA	A/Changsha/1000/2022	EPI2035836
PA	A/chicken/Anhui/LH99/2017	MH489450.1
PA	A/Chicken/Beijing/1/94	AF156466
PA	A/Chicken/Beijing/1/94	AF156480
PA	A/chicken/China/93/2017	MN385408.1
PA	A/chicken/China/Guangdong_01/2022	ON626404.1
PA	A/chicken/China/H1072/2017	MN100439.1
PA	A/chicken/Dubai/338/2001	EPI110388
PA	A/chicken/Fujian/1.25_FZHX0003-O/2018	MW102847.1
PA	A/chicken/Guangdong/1.25_SZBJ014-O/2018	MW096458.1
PA	A/chicken/Guangxi/DX/2008	EPI610280
PA	A/chicken/Guizhou/06.30_ZYLJJ002-O/2018	MW104255.1
PA	A/chicken/Hunan/6.27_YYGK57S2-OC/2017	MW105183.1
PA	A/chicken/Jiangxi/6.26_NCDZT48R2-OC/2017	MW106487.1
PA	A/chicken/Nanjing/B854-2/2011	KU158925
PA	A/chicken/Neimenggu/NMG7/2018	OM019053.1
PA	A/chicken/Qingyuan/zd201602/2016	MK250032.1
PA	A/chicken/Shandong/01.26_TAWL012-O/2019	MW106711.1
PA	A/Chicken/Shandong/6/96	EPI82044
PA	A/chicken/Shandong/98/2018	MW389301.1
PA	A/chicken/Tajikistan/2379/2018	MW786968.1
PA	A/chicken/Xinjiang/3.18_WLMQXL023-C/2017	MW096521.1
PA	A/chicken/Zhejiang/HJ/2007	EPI221854
PA	A/duck/Guangdong/8.30_DGCP06-O/2017	MW096611.1

Gene	Identification	GISAID accession no.
PA	A/Duck/Hong Kong/Y280/97	AF156405
PA	A/Duck/Hong Kong/Y280/97	AF156461
PA	A/Duck/Hong_Kong/Y439/97	EPI6029
PA	A/duck/Hunan/6.27_YYGK65S2-OC/2017	MW108455.1
PA	A/duck/Jiangxi/1.31_NCNP32I3-OC/2018	MW096283.1
PA	A/duck/Nanjing/A1591-1/2010	KU158924
PA	A/Environment/Jiangxi/14737/2014	EPI858162
PA	A/environment/Shandong/1.26_TAWL004-E/2019	MW109775.1
PA	A/Guangdong/HP001/2017	KY643841.1
PA	A/Guangdong/W1/2004	EPI846046
PA	A/Henan/4-10CNIC/2022	EPI2026166
PA	A/Henan/4-14CNIC/2022	EPI2026174
PA	A/Hong_Kong/3239/2008	CY055155.1
PA	A/Hunan/44558/2015	EPI680522
PA	A/Jiangxi/1/2013	KM392410.1
PA	A/Quail/Hong Kong/G1/97	AF156463
PB1	A/Anhui-Lujiang/39/2018	EPI1315824
PB1	A/Beijing/1/2013	KJ476631.1
PB1	A/Changsha/1000/2022	EPI2035835
PB1	A/chicken/Beijing/0331/2013	KM609837.1
PB1	A/Chicken/Beijing/1/94	AF156452
PB1	A/chicken/China/795/2014	MK446866.1
PB1	A/chicken/China/Guangdong_01/2022	ON626405.1
PB1	A/chicken/Dubai/338/2001	EPI110349
PB1	A/chicken/Fujian/MQ01/2015	MT774534.1
PB1	A/chicken/Guangxi/DX/2008	EPI610273
PB1	A/Chicken/Hong Kong/G9/97	AF156402
PB1	A/Chicken/Hong Kong/G9/97	AF156458
PB1	A/chicken/Jinan/3952/2013	KP415266.1
PB1	A/chicken/Juxian/3387/2013	KP415242.1
PB1	A/chicken/Nanjing/B854-2/2011	KU158932
PB1	A/chicken/Rizhao/515/2013	KF260709.1
PB1	A/Chicken/Shandong/6/96	EPI82098
PB1	A/chicken/Shaoxing/5088/2013	KP417192.1
PB1	A/chicken/Suzhou/040201H/2013	KM879363.1
PB1	A/chicken/Wuxi/0405005/2013	KT779599.1
PB1	A/chicken/Zhejiang/HJ/2007	EPI221859
PB1	A/chicken/Zhejiang/S1190/2015	MF630363.1
PB1	A/Duck/China/F1206/2015	MN100268.1
PB1	A/Duck/Hong Kong/Y280/97	AF156447
PB1	A/Duck/Hong_Kong/Y439/97	EPI5973
PB1	A/duck/Nanjing/A1591-1/2010	KU158931
PB1	A/Duck/Shandong/Y2/2017	MH375437.1
PB1	A/Environment/Jiangxi/14737/2014	EPI858164
PB1	A/environment/Jilin/04.11_CCHSL006-E/2018	MW109726.1
PB1	A/Guangdong/W1/2004	EPI846045
PB1	A/Hangzhou/1/2013	KF001508.1
PB1	A/Henan/4-10CNIC/2022	EPI2026168
PB1	A/Henan/4-14CNIC/2022	EPI2026176
PB1	A/Hong_Kong/3239/2008	CY055154.1
PB1	A/Hunan/44558/2015	EPI680524
PB1	A/Jiangxi/1/2013	KM392409.1
PB1	A/mink/Shandong/Z1/2015	KY272073.1
PB1	A/pigeon/Jiangsu/K113/2013	KP185908.1
PB1	A/Quail/Hong Kong/G1/97	AF156449
PB1	A/quail/Zhejiang/2A6/2013	KU042150.1
PB1	A/silkie chicken/Shaoxing/2471/2013	KP415311.1
PB1	A/wild chicken/Shanghai/C1/2014	KJ726730.1
PB2	A/Anhui-Lujiang/39/2018	EPI1315823
PB2	A/Anser fabalis/Anhui/L139/2014	KT699053.1
PB2	A/Anser fabalis/China/HuBS428/2014	KM076701.1
PB2	A/Changsha/1000/2022	EPI2035834
PB2	A/Chicken/Beijing/1/94	AF156438.1
PB2	A/chicken/China/828/2015	MK446792.1
PB2	A/chicken/China/Guangdong_01/2022	ON626406.1
PB2	A/chicken/Dongguan/1674/2014	KP416443.1
PB2	A/chicken/Dubai/338/2001	EPI110402
PB2	A/chicken/Guangdong/SD027/2017	MF630106.1

Gene	Identification	GISAID accession no.
PB2	A/chicken/Guangxi/DX/2008	EPI610278
PB2	A/Chicken/Hong Kong/G9/97	AF156430.1
PB2	A/chicken/Hubei/S0485/2015	MN647243.1
PB2	A/chicken/Jiangxi/29086/2013	KP285282.1
PB2	A/chicken/Nanjing/B854-2/2011	KU158939
PB2	A/Chicken/Shandong/6/96	EPI82152
PB2	A/Chicken/Shanghai/F/98	AY253750
PB2	A/chicken/Shantou/4832/2014	KP418213.1
PB2	A/chicken/Suzhou/4837/2013	KP414747.1
PB2	A/chicken/Zhejiang/HJ/2007	EPI221853
PB2	A/chicken/Zhejiang/SC324/2013	KM113058.1
PB2	A/Duck/China/F3143/2015	MN099548.1
PB2	A/Duck/Hong Kong/Y280/97	AF156433.1
PB2	A/Duck/Hong_Kong/Y439/97	EPI6001
PB2	A/duck/Hubei/S1035/2014	MN647475.1
PB2	A/duck/Nanjing/A1591-1/2010	KU158938
PB2	A/duck/Wuhan/WHYF14/2014	KU143587.1
PB2	A/enviroment/Hubei/S0879/2014	MN647491.1
PB2	A/environment/Hunan/27420/2014	KT356726.1
PB2	A/Environment/Jiangxi/14737/2014	EPI858163
PB2	A/Guangdong/W1/2004	EPI846044
PB2	A/Henan/4-10CNIC/2022	EPI2026167
PB2	A/Henan/4-14CNIC/2022	EPI2026175
PB2	A/Hong_Kong/3239/2008	CY055153.1
PB2	A/Hunan/44558/2015	EPI680523
PB2	A/Jiangxi/1/2013	KM392408.1
PB2	A/Quail/Hong Kong/G1/97	AF156435.1
PB2	A/wild birds/Hubei/45/2014	MH991752.1
PB2	A/WuXi/0126/2014	MG214182.1

*HA, hemagglutinin; M, matrix; NA, neuraminidase; NP, nucleoprotein, NS, nonstructural protein; PA, polymerase acidic; PB, polymerase basic.

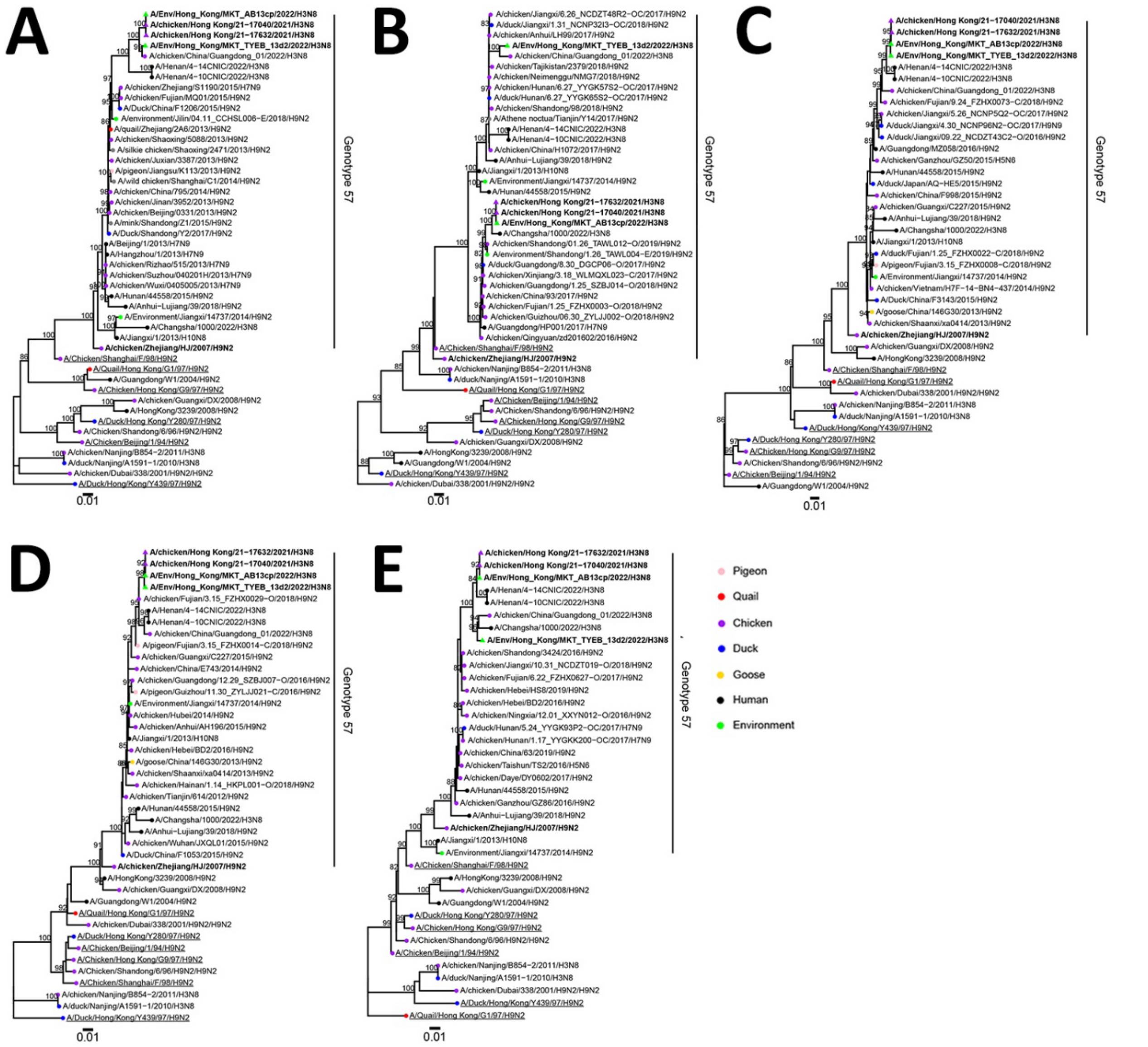

Appendix Figure. Phylogenetic analysis of influenza A(H3N8) viruses isolated from chicken farms, live poultry markets, and the Mai Po Wetlands, Hong Kong, China. Viruses listed in Table 5 were analyzed with other relevant virus sequence data available in public data bases (accession numbers in Appendix Table). Trees were generated by using IQ-tree (www.iqtree.org) with the general time reversible + gamma model. Bootstrap values ≥80% are shown. Scale bars indicate estimated genetic distance. Influenza A(H3N8) viruses isolated from chicken farms, live poultry markets, and the Mai Po Wetlands in Hong Kong are indicated in red. Bold indicates sequences isolated in this study. A) Polymerase basic 1 gene segment; B) polymerase acidic gene segment; C) nucleoprotein gene segment; D) matrix gene segment; E) nonstructural protein gene segment.
